# Development of novel *Escherichia coli* cell-based biosensors to monitor Mn(II) in environmental systems

**DOI:** 10.3389/fmicb.2022.1051926

**Published:** 2022-12-19

**Authors:** Yangwon Jeon, Yejin Lee, Yeonhong Kim, Chanhee Park, Hoon Choi, Geupil Jang, Youngdae Yoon

**Affiliations:** ^1^Department of Environmental Health Science, Konkuk University, Seoul, Republic of Korea; ^2^Department of Life and Environmental Sciences, Wonkwang University, Iksan, Republic of Korea; ^3^School of Biological Sciences and Technology, Chonnam National University, Gwangju, Republic of Korea

**Keywords:** *E. coli*-based biosensor, Mn(II) monitoring, MntR transcription factor, *mnt*-operon, mancozeb

## Abstract

*Escherichia coli* uses manganese [Mn(II)] as an essential trace element; thus, it has a genetic system that regulates cellular Mn(II) levels. Several genes in the *mnt*-operon of *E. coli* respond to intercellular Mn(II) levels, and transcription is regulated by a transcription factor (MntR) that interacts with Mn(II). This study aimed to develop Mn(II)-sensing biosensors based on *mnt*-operon genetic systems. Additionally, the properties of biosensors developed based on the promoter regions of *mntS*, *mntH*, and *mntP* were investigated. MntR represses the transcription of MntS and MntH after binding with Mn(II), while it induces MntP transcription. Thus, Mn(II) biosensors that decrease and increase signals could be obtained by fusing the promoter regions of *mntS/mntH* and *mntP*, with *egfp* encoding an enhanced green fluorescent protein. However, only the biosensor-based *mntS:egfp* responded to Mn(II) exposure. Further, *E. coli* harboring *P_*mntS*_:egfp* showed a concentration-dependent decrease in fluorescence signals. To enhance the sensitivity of the biosensor toward Mn(II), *E. coli* containing a deleted MntP gene that encodes Mn(II) exporter, was used as a host cell for biosensor development. The sensitivity toward Mn(II) increased by two times on using *E. coli-mntP*, and the biosensor could quantify 0.01–10 μM of Mn(II). Further, the applicability of Mn(II) in artificially contaminated water samples was quantified and showed >95% accuracy. The newly developed Mn(II) biosensors could detect and quantify the residual Mn(II) from mancozeb in soil samples, with the quantification accuracy being approximately 90%. To the best of our knowledge, this is the first Mn (II)-specific bacterial cell-based biosensor that serves as a valuable tool for monitoring and assessing the risks of Mn(II) in environmental systems.

## 1 Introduction

Manganese is a trace element essential for living organisms, and its deficiency can cause numerous biochemical and structural abnormalities (Keen et al., 1999; Dobson et al., 2004). It is required for normal development, maintenance of nerve and immune cell functions, and regulation of blood sugar and vitamins (Keen et al., 2000; Liu et al., 2013). Moreover, Mn serves as a cofactor for diverse enzymes, and nucleotide metabolism and signaling, and is an important protectant against oxidative stresses (Vieira et al., 2012; Aliko et al., 2018). However, overexposure of Mn causes toxicity in living organisms, including humans (Crossgrove and Zheng, 2004; O’Neal and Zheng, 2015). Compared to other toxic heavy metals, Mn has not been studied extensively because of its low toxicity and low application rates. However, the use of Mn has increased recently in diverse industrial fields (Qu et al., 2013). For example, energy production has shifted to sustainable and green methods; moreover, the demand of Mn in rechargeable batteries as Mn-based oxides has increased immensely worldwide (Kim and Manthiram, 1997; Zhao et al., 2019). Recent studies have reported that Mn accumulation caused by Mn(II)-conjugated pesticides and fungicides, which are widely used in agricultural sectors, adversely affects human health (De Joode et al., 2016; Costa-Silva et al., 2018). However, although Mn(II) can cause serious environmental pollution and adverse health effects, it has not received considerable research attention.

Similar to other heavy metals, Mn can be quantified and analyzed using instrument-based analytical techniques, such as inductively coupled plasma (ICP)–atomic emission spectroscopy (AES) and ICP–mass spectroscopy. Although such techniques have advantages of high precision and accuracy, they are time-consuming and expensive. Accordingly, several different sensors, such as electrochemical, nanomaterial, chemical, and bacterial cell-based biosensors, have been developed based on novel techniques to detect heavy metal(loid)s (Kim et al., 2020). All these sensors exhibit certain advantages and disadvantages; particularly, bacterial cell-based biosensors, also known as whole-cell bioreporters, have been actively investigated to monitor heavy metals (Magrisso et al., 2008; Hynninen and Virta, 2009; Van Der Meer and Belkin, 2010). Bacterial cell-based biosensors share common components, such as sensing domains and reporter domains derived from stress-responsive genetic systems and gene-encoding fluorescent proteins or enzymes, respectively. If appropriate genetic systems corresponding to different targets are available, target-sensing bacterial cell-based biosensors can be obtained (Elcin et al., 2021; Henshke et al., 2021; Zappi et al., 2021). In fact, various heavy metal(loid)-sensing biosensors have been developed based on the available genetic systems; however, Mn-targeted biosensors have not yet been reported. Although Mn is relatively less toxic than other hazardous metals, it is still hazardous to animals and plants at high concentrations (Reaney et al., 2002; Crossgrove and Zheng, 2004). As the use of Mn is expected to increase in the next generation energy era, developing a bacterial cell-based biosensor to detect and monitor Mn in environmental systems is important.

Previous studies have identified genes associated with Mn(II) homeostasis, and reported that MntR acts as a regulatory protein at the Mn(II) level in *Escherichia coli* (Guedon and Helmann, 2003). Moreover, MntR represses the transcription of *mntH* and *mntS*, an Mn(II) importer and a small uncharacterized protein, respectively, and activates *mntP*, an Mn(II) exporter, in the presence of Mn(II). Although the role of MntS was not clearly characterized, it was speculated as an inhibitor of MntP to maintain cellular Mn level constant (Waters et al., 2011; Martin et al., 2015). In other words, MntH, MntS, and MntP regulate Mn(II) homeostasis in *E. coli via* the functions of MntR, a regulatory protein in the Mn(II)-responsive operon. Therefore, the Mn(II)-responsive genetic system could be employed to generate Mn(II)-specific bacterial cell-based biosensors. Since *mntS/mntH* and *mntP* are contrastingly regulated by MntR, the promoters of these two genes could be used as genetic systems for developing Mn(II) biosensors that increase and decrease signals, respectively.

In this study, we developed Mn(II)-sensing biosensors based on *mnt*-operon genetic systems. Biosensors based on the promoter regions of *mntS*, *mntH*, and *mntP* were generated, and their properties were investigated. Additionally, the MntP gene encoding Mn(II) exporter was deleted in the host cells to enhance sensitivity of the biosensor toward Mn(II), and point mutations were introduced in MntR to modify the target-sensing properties. Finally, the applicability of the newly developed Mn(II) biosensors was tested by quantifying Mn(II) concentrations in artificially contaminated water and soil samples. To the best of our knowledge, this study is the first to report a Mn(II)-sensing biosensor based on *E. coli* cells.

## 2 Materials and methods

### 2.1 Materials

*Escherichia coli* BL21 and DH5α were used as host strains for biosensor development and plasmid construction. The heavy metals (Mn, As, Cd, Ni, Hg, Pb, Au, Sb, Co, Fe, Zn, and Cr) used in this study were purchased from Sigma Aldrich (Steinheim, Germany) in chloride salt form and were prepared as 1 mM stocks. All restriction enzymes, namely, *Nde*I, *Bgl*II, *Bam*HI, *Xba*I, *Xho*I, and DNA T4 ligase, were purchased from Takara, Korea. Taq polymerase used for gene amplification was purchased from Takara Biomedical, and Turbu Pfu used for site-directed mutagenesis was purchased from New England Biolabs. Primer synthesis and DNA sequencing were performed by Macrogen (Seoul, South Korea). Mancozeb, which was used to prepare artificially contaminated samples, was purchased from Sigma-Aldrich.

### 2.2 Plasmid constructions

The promoter regions of *mntP, mntH*, and *mntS* were amplified by polymerase chain reaction (PCR) from the genomic DNA of *E. coli* BL21 and were inserted into pET21-eGFP with *Bgl*II and *Xba*I restriction sites. The plasmids carrying the promoter regions and fluorescence protein genes were named pMntS-eGFP, pMntH-eGFP, and pMntP-eGFP. Additionally, *mntP*-riboswitch:*egfp* was fused because the riboswitch region is related to *mntP* transcription (Patzer and Hantke, 2001; Waters et al., 2011). Further, the MntR-encoding gene was amplified from the genomic DNA and inserted into pCDF-Duet with *Nde*I and *Xho*I to generate pCDF-MntR. MntR mutants were obtained by site-directed mutagenesis using corresponding primer sets. All the plasmids constructed in this study were confirmed using DNA sequencing. Table 1 lists the *E. coli* strains and plasmids used in this study, while Supplementary Table 1 lists the primers used for plasmid construction.

**TABLE 1:** The lists of *Escherichia coli* strains and plasmids used in this study.

	Name	Description	References
Strains	*E. coli* BL21(DE3) *E. coli-mntR* *E. coli-mntP* *E. coli-mntR/mntP*	F^–^ *ompT hsdS_*B*_*(r_B_^–^m_B_^–^)*gal dcm lon* (DE3) *E. coli* BL21 Δ*mntR:FRT* *E. coli* BL21 Δ*mntP:FRT* *E. coli* BL21 Δ*mntR/*Δ*mntP:FRT*	Stratagene This study This study This study
Plasmids	pET21(a) pCDF-Duet pMntS-eGFP pMntP-eGFP pMntP-ribo-eGFP pMntH-eGFP	pBR322 ori, Amp^r^ CloDE13 ori, Str^r^ pET21(a) carrying P*_*mntS*_*:egfp pET21(a) carrying P*_*mntP*_*:egfp pET21(a) carrying P*_*mntP*_*-riboswitch:egfp pET21(a) carrying P*_*mntH*_*:egfp	This study Novagen Novagen This study This study This study
	pCDF-MntR WT pCDF-MntR K72L pCDF-MntR R77L pCDF-MntR H135A	pCDF-Duet carrying *mntR* WT and mutants	This study

### 2.3 Gene deletion in *E. coli*

The endogenous genes, *mntP* and *mntR* that encoded MntP and MntR, respectively, in *E. coli* BL21 were deleted using the Quick and Easy *E. coli* Gene Deletion Kit, following the manufacturer’s instructions with minor modifications. The FLP recognition target (FRT)-flanked PGK-gb2-neo cassette with target genes were amplified and introduced into *E. coli* BL21(DE3) cells harboring the pRedET plasmid, which carried the gene encoding recombinase, to replace entire open reading frames of mntP and mntR (Kang et al., 2018b). The target genes were replaced with the kanamycin resistance gene (*kan*) by adding 10% arabinose solution. After the *kan* gene in single-deletion *E. coli* was deleted, the same process was repeated to generate a double-gene-deleted *E. coli* strain. The deletion of *mntR*, *mntP*, and *mntR/mntP* was further confirmed by PCR, and the corresponding gene-deficient *E. coli* strains were referred to as *E. coli-mntR*, *E. coli-mntP*, and *E. coli-mntR/mntP*, respectively.

### 2.4 Biosensor assays

*Escherichia coli* cell-based biosensors were generated by transforming the reporter plasmids, including pMntS-eGFP, pMntH-eGFP, pMntP-eGFP, and pMntP-ribo-eGFP, into *E. coli* BL21 WT and mutant strains. pCDF-MntR was introduced into the *mntR*-deficient strains, *E. coli-mntR* and *E. coli-mntR/mntP*. Several *E. coli* cell-based biosensors generated by combining plasmids and strains were applied in biosensor assays to verify their sensing properties. To conduct the biosensor assays, *E. coli* cell-based biosensors were grown overnight and then inoculated with fresh Luria-Bertani (LB) media. The cells were grown until the optical density at 600 nm (OD_600_) reached 0.3; subsequently, the cells were exposed to 0–10 μM of the tested heavy metal(loid) ions, and the expression level of enhanced green fluorescence protein (eGFP) was measured using a fluorescence spectrophotometer (FluoroMate FS-2, Scinco, Seoul, South Korea) after an incubation period of 2 h. To evaluate the effects of MntR mutations and MntP deletions, gene-deficient *E. coli* strains were used as host cells for biosensors, and a biosensor assay was performed. The biosensors were exposed to different metal(loid) ions concentrations, and the expression level of eGFP was measured at different periods. The expression level of eGFP was measured using a fluorescence spectrophotometer equipped with a bandwidth of 1 nm for filters, and 480 nm/520 nm wavelengths were used for excitation/emission. The harvested cells were resuspended in 50 mM Tris buffer (pH 7.4) containing 160 mM HCl to remove LB before conducting analysis using the fluorescence spectrophotometer. Finally, to consider the toxic effects of heavy metals on the growth of *E. coli*, the arbitrary fluorescent signals induced by heavy metal exposure were divided by the OD_600_ values.

### 2.5 Mn(II) quantifications in artificially contaminated samples

To investigate the applicability of *E. coli* cell-based biosensors, Mn(II) was quantified in artificially contaminated samples using the developed biosensors. Artificially contaminated water samples were prepared by spiking known concentrations of Mn(II) and manganese zinc ethylenebis (mancozeb), while artificially contaminated soil samples were prepared by treating 1 g of loam soil with different amounts of mancozeb. The leachate was then prepared by adding 10 mL of sterilized water and centrifuging at 37°C at 250 rpm for 3 days. The artificially contaminated soil and water samples were applied to *E. coli* cell-based biosensors, and residual Mn(II) was determined using a biosensor assay. To compare the accuracy of the biosensor assay, the residual Mn(II) in the leachates of the soil samples was analyzed using ICP–AES by the Korea Basic Science Institute.

### 2.6 Data analysis

R 4.1.0 and DescTools package were used for the statistical analysis of the data when the minimum difference between the control and treatment means was statistically significant (R Core Team, 2014; Signorell et al., 2019). All experimental data was obtained from more than triplicated independent experiments.

## 3 Results

### 3.1 Mn(II)-sensing *E. coli* cell-based biosensors

**FIGURE 1:**
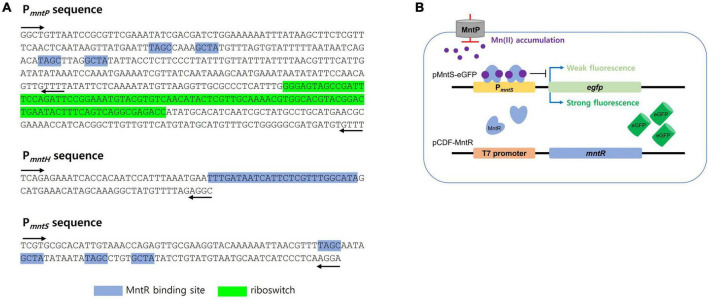
DNA sequence of promoter regions and the diagram of Mn(II)-sensing *Escherichia coli* cell-based biosensors. **(A)** DNA sequences of the promoter regions of *mntP, mntH*, *and mntS*. MntR binding sites and the riboswitch region was indicted blue and green highlight, respectively. The black arrows indicated the locations of primers for cloning. **(B)** The diagram for Mn(II)-sensing *E. coli* cell-based biosensor harboring pMntS-eGFP. MntR was not tightly bound to P*_*mntS*_* without Mn(II) to induce strong fluorescence, while the signal was weakened with Mn(II) by tight binding of MntR.

**FIGURE 2:**
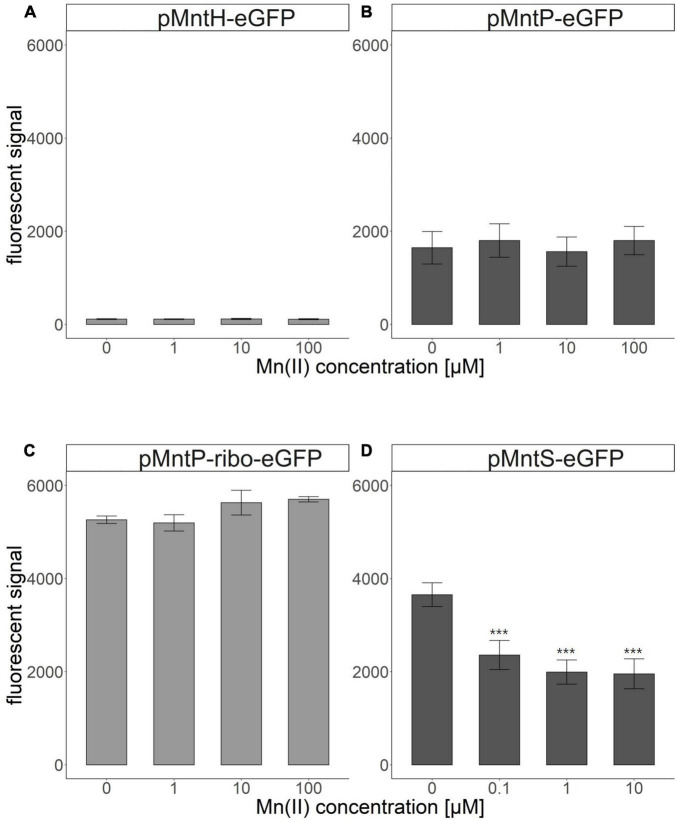
Fluorescence signals of *Escherichia coli* cell-based biosensors upon Mn(II) exposure. **(A)** *E. coli* harboring pMntH-eGFP, **(B)** *E. coli* harboring pMntP-eGFP, **(C)** *E. coli* harboring pMntP-ribo-eGFP, and **(D)** *E. coli* harboring pMntS-eGFP. The data was obtained from more than three times of replicated experiments. The asterisks indicate that the data is significantly higher than the control data (Dunnett’s test, ***p < 0.001).

The promoter regions of *mntS, mntP*, and *mntH* in the *mnt*-operon of *E. coli* are related to Mn homeostasis, and their transcription levels are controlled by the amount of endogenous Mn(II). Thus, these promoter regions were fused with *egfp* to construct pMntS-eGFP, pMntH-eGFP, pMntP-eGFP, and pMntP-ribo-eGFP plasmids. In case of the promoter of *mntP*, pMntP-eGFP, and pMntP-ribo-eGFP were tested because the riboswitch region between P*_*mntP*_* and *mntP* was known to play a role in the transcription of *mntP* in the previous reports (Patzer and Hantke, 2001; Sun et al., 2012). The sequences of P*_*mntP*_*, P*_*mntH*_*, and P*_*mntS*_* are shown in Figure 1A, and the working mechanism of the biosensor is shown in Figure 1B. As gene transcription under these promoters was expected to respond to Mn(II), *E. coli* BL21 (DE3) cells harboring these plasmids were exposed to different concentrations of Mn(II) for 2 h during the biosensor assay. The changes in the expression levels of eGFP were investigated using the fluorescence spectrophotometer. Except for pMntS-eGFP, others showed no significant changes in the expression levels after Mn(II) exposure. The biosensor-based pMntH-eGFP did not induce eGFP expression, even after exposure to 100 μM Mn(II) (Figure 2A). Contrastingly, eGFPs under P*_*mntP*_* were expressed in the biosensors that employed pMntP-eGFP and pMntP-ribo-eGFP irrespective of Mn(II) concentrations (Figures 2B, C). Although alleviated eGFP signals were observed through the biosensors based on both pMntP-eGFP and pMntP-ribo-eGFP, the signal changes after 0–100 μM of Mn(II) exposure were not sufficient for Mn(II) biosensors. On the other hand, the *E. coli* cell-based biosensor harboring pMntS-eGFP showed a decreased eGFP signal upon Mn(II) exposure (Figure 2D). This result was concordant with our expectations because MntS is repressed by high levels of endogenous Mn(II) (Martin et al., 2015). This was because MntR acts as a transcription factor (TF), and high levels of Mn(II) trigger the Mn(II)-bound MntR located in the P*_*mntS*_* region, resulting in the repression of eGFP expression (Figure 1B). Additionally, the *E. coli* harboring promoterless eGFP was tested as a negative control and no fluorescence was observed (data not shown).

**FIGURE 3:**
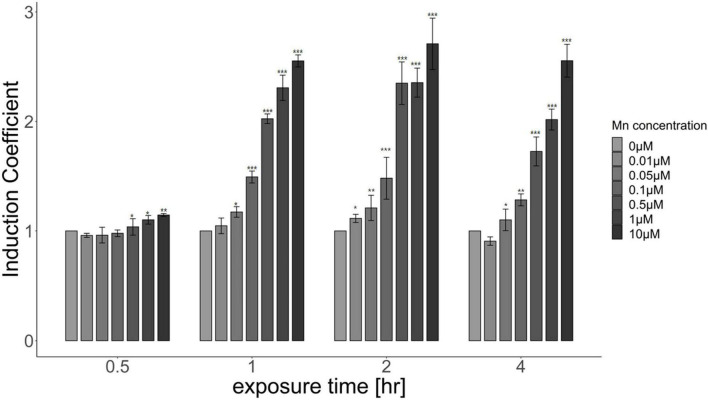
Responses of *Escherichia coli* cell-based biosensors harboring pMntS-eGFP upon Mn(II) exposure. The biosensors were exposed to different Mn(II) concentrations, and the induction coefficient values were measured after 0.5, 1, 2, and 4 h of exposure. The data was obtained from more than three times of replicated experiments. The asterisks indicate that the treatment values were higher than the control values (Dunnett’s test, *p < 0.05, **p < 0.01, and ***p < 0.001).

Because the biosensor based on pMntS-eGFP responded to Mn(II) concentration, it was employed for further investigation to characterize the properties of Mn(II)-sensing biosensors. However, optimizing the experimental conditions was necessary to achieve optimum biosensor performance. *Escherichia coli* cell-based biosensors harboring pMntS-eGFP were exposed to 0–10 μM Mn(II), and the fluorescence intensity was measured for up to 4 h. The changes in fluorescence intensity were converted to induction coefficient values as follows: [eGFP signal without Mn(II) exposure]/[eGFP signal with Mn(II) exposure]. As shown in Figure 3, the changes in the fluorescence intensity increased with the Mn(II) concentration after 1 h, and significant signal changes were observed after 2 h of exposure of 0.5 and 1 μM Mn(II). Additionally, it was noticed that the newly developed Mn(II) biosensor showed the capability to detect 0.05 and 0.1 μM Mn(II).

### 3.2 MntR regulates the expression of enhanced green fluorescence protein under P_*mntS*_

**FIGURE 4:**
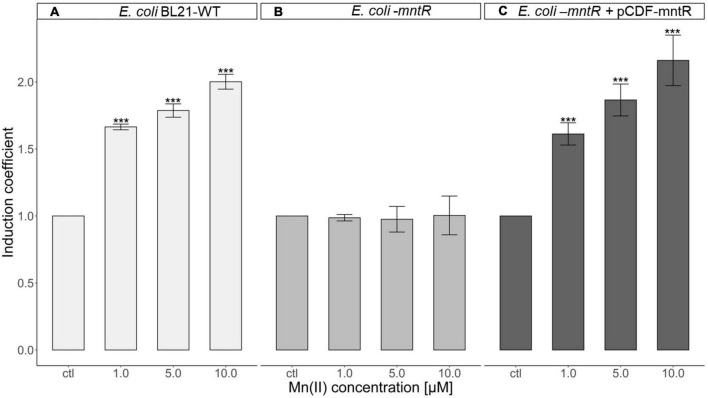
Role of MntR in Mn(II)-sensing *Escherichia coli* cell-based biosensor harboring pMntS-eGFP. **(A)** Mn(II) response of *E. coli* WT harboring pMntS-eGFP, **(B)**, *E. coli-mntR* harboring pMntS-eGFP, and **(C)** *E. coli-mntR* harboring pMntS-eGFP/pCDF-MntR WT. The repression of *egfp* under P*_*mntS*_* was prevented by *mntR* deletion and restored by introducing recombinant MntR. The biosensors were exposed to Mn(II) for 2 h. The data was obtained from more than three times of replicated experiments. The asterisks indicate that the treatment values were higher than the control values (Dunnett’s test, ***p < 0.001).

To verify the effects of MntR, *mntR*-deficient *E. coli* was developed and used as a host strain. The plasmids, pMntS-eGFP and pCDF-MntR, were transformed into *E. coli* WT and *E. coli-mntR* to generate *E. coli* WT_pMntS-eGFP, *E. coli-mntR*_pMntS-eGFP, and *E. coli-mntR*_pMntS-eGFP/pCDF-MntR. The *E. coli* WT with pMntS-eGFP showed a weak green signal under ultraviolet light because of the presence of MntR. However, the green signal was strong for *E. coli-mntR*, while it decreased again when recombinant MntR was introduced into the *mntR*-deficient *E. coli* (Supplementary Figure 1). Although it was not clear how MntR regulates the expression MntS, the presence of MntR represses mntS in *E. coli*. Moreover, the biosensors, including pMntS-eGFP in *mntR*-deficient *E. coli* and *E. coli* WT, and pMntS-eGFP and pCDF-MntR in *mntR*-deficient *E. coli*, were exposed to 0–10 μM of Mn(II), and the eGFP signal was indicated as induction coefficient values (Figure 4). The biosensor based on *E. coli* WT showed the changes in eGFP signals, while the biosensor without MntR was not responded to Mn(II) exposure (Figures 4A, B). Then, the signal changes were recovered from the recombinant MntR was introduced to *E. coli-mntR* (Figure 4C). This was concordance to the result shown in Supplementary Figure 1 and would be another evidence MntR plays a role in MntS expression. Although biosensors based on pMntS-eGFP showed signals in Mn(II) concentration dependent manner, the sensitivity to Mn(II) was insufficient because about two times of increase in the signal changes was observed at 10 μM. Thus, the further investigation was necessary to enhance the sensitivity to Mn(II).

### 3.3 Intracellular accumulation of Mn(II) in mntP-deficient *E. coli*

**FIGURE 5:**
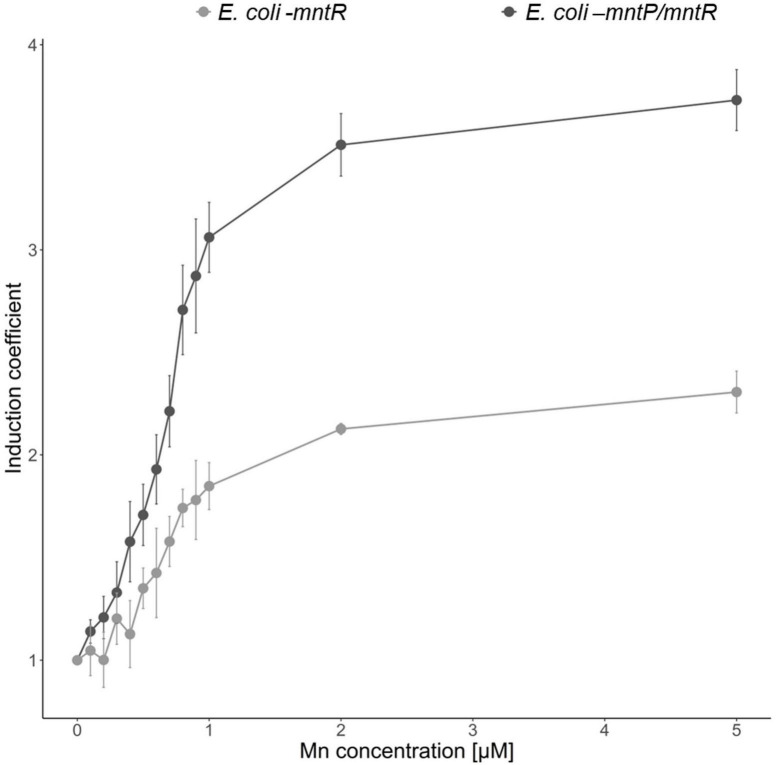
Effects of MntP on Mn(II) sensitivity of *Escherichia coli* cell-based biosensors. *E. coli-mntR* (gray) and *E. coli-mntR/mntP* (black) harboring pMntS-eGFP/pCDF-MntR were exposed to Mn(II), and the corresponding fluorescence signals were compared after 2 h. The data was obtained from more than three times of replicated experiments.

An increase in intracellular Mn(II) induces the expression of MntP, a Mn(II) exporter, to keep manganese concentration constant (Martin et al., 2015). In this aspect, the accumulation of intracellular Mn(II) would be expected if MntP was deleted. To test the effects of MntP on the Mn(II)-sensing properties, *E. coli-mntR/mntP* was generated by deleting *mntP* in *mntR*-deficient *E. coli* and used as a host strain. pMntS-eGFP and pCDF-MntR were transformed into *E. coli-mntR* and *E. coli-mntR/mntP*, and the corresponding expression levels of eGFP were compared. The biosensors were exposed to 0–5 μM Mn(II), and the induction coefficient values were obtained after 2 h of incubation. The levels of eGFP decreased with increasing Mn(II) concentrations, and the *mntP*-deficient strains showed enhanced responses to Mn(II) (Figure 5). *Escherichia coli* with *mntP* showed approximately 2.0 of induction coefficient values after exposure to 2 and 5 μM Mn(II), while *E. coli* without *mntP* showed approximately two-times enhanced signal changes. Thus, we inferred that the *mntP* deletion accumulated intracellular Mn(II) because the two strains responded differently to the same Mn(II) concentration. Moreover, the sensitivity of the biosensors could be enhanced by genetic engineering of host cells, and MntP, a Mn(II) exporter, was related to the removal of intracellular Mn(II) from cells.

### 3.4 Mn(II)-specific *E. coli* cell-based biosensor

**FIGURE 6:**
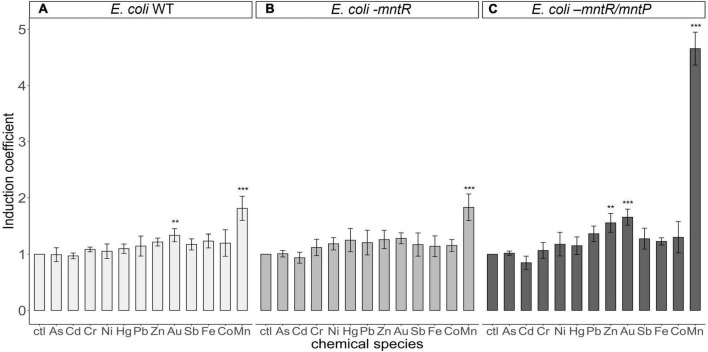
Metal(loid) selectivity of biosensors harboring pMntS-eGFP and pCDF-MntR based on host strains. The fluorescence signals of biosensors based on **(A)** *E. coli* BL21 WT, **(B)** *Escherichia coli-mntR*, and **(C)** *E. coli-mntP/mntR* as host strains were measured after 2 h of exposure to 5 μM of different heavy metal(loid)s. The data was obtained from more than three times of replicated experiments. The differences in the fluorescence signals among the heavy metal(loid)s were compared using analysis of variance with *post hoc* Tukey’s test (**p < 0.01 and ***p < 0.001).

As transcription under P*_*mntS*_* was regulated by MntR, the target specificity of biosensors was determined by the target specificity of MntR. Thus, understanding whether the changes in eGFP expression were stimulated by heavy metal(loid)s other than Mn(II) was necessary. Accordingly, verifying the selectivity of the newly generated biosensors was also essential. The biosensors, *E. coli* BL21 harboring pMntS-eGFP, *E. coli-mntR* with pMntS-eGFP and pCDF-MntR, and *E. coli-mntR/mntP* with pMntS-eGFP and pCDF-MntR were exposed to 5 μM of other heavy metal(loid) ions, namely, As, Cd, Cr, Ni, Hg, Pb, Zn, Au, Sb, Fe, Co, and Mn for 2 h, after which the expression level of eGFP was determined (Figure 6). Notably, all tested biosensors responded only to Mn(II) exposure. Moreover, *mntP* deletion considerably enhanced the sensitivity of the biosensor to Mn(II). When the host strains exhibited MntP, the signal changes after Mn(II) exposure were less by two times, while the signals of *E. coli-mntR/mntP* increased approximately five times. However, compared with other biosensors, *E. coli-mntR/mntP* showed weak responses toward Au, Zn, and Pb. The biosensor was exposed to different concentrations of those heavy metals, but no concentration dependent response was observed (data not shown). Therefore, it was concluded that the new biosensor based on *E. coli-mntR/mntP* harboring pMntS-eGFP/pCDF-MntR was highly specific and selective to Mn(II).

### 3.5 Applicability of Mn(II)-specific *E. coli* cell-based biosensor

As described previously, the biosensor with pMntS-eGFP showed specific responses toward Mn(II), and the sensitivity toward Mn(II) was enhanced using *E. coli-mntR/mntP* as a host strain with pMntS-eGFP and pCDF-MntR. Thus, evaluating the applicability of new biosensors is necessary. To test its applicability, the concentration of Mn(II) in the artificially contaminated water and soil samples was quantified using a biosensor assay. The Mn(II) contaminated samples were prepared by spiking MnCl_2_ and Mn(II)-conjugated fungicide, mancozeb. Since the mancozeb is one of most used fungicides and releases Mn(II) to environmental systems, it was chosen to show the applicability of new Mn(II) specific biosensor.

In the case of water samples, the Mn(II)-specific biosensors were subjected to 1.2, 1.4, 1.6, and 1.8 μM Mn(II)-spiked samples, and the amount of Mn(II) was calculated from a standard curve obtained using 0–5 μM Mn(II). On comparing the Mn(II) concentrations determined by the biosensors with the original spiked concentrations (Table 2), the biosensor assay accuracy was found to be approximately 95%. Similar to the Mn(II)-spiked water samples, 1.0, 10.0, and 50.0 μM mancozeb was spiked for the biosensors from 1 mM mancozeb solution. The signal changes after mancozeb treatment were determined using a biosensor assay, and the amount of Mn(II) released from mancozeb was calculated using a standard curve (Table 3). Consequently, 2.00 ± 0.55, 6.08, and 9.62 μM of Mn(II) was detected from 1.0, 10.0, and 50.0 μM of mancozeb exposure, respectively. The amount of Mn(II) was not proportional to the mancozeb amount, possibly due to the degradation rate and purity of the purchased mancozeb.

**TABLE 2:** The quantification of Mn(II) in artificially contaminated water samples.

Spiked Mn(II) (μM)	Determined concentration (μM)	Accuracy (%)
1.2	1.34 ± 0.094	90.0
1.4	1.47 ± 0.030	95.3
1.6	1.63 ± 0.054	98.4
1.8	1.89 ± 0.091	95.4

**TABLE 3:** The quantification of Mn(II) in mancozeb spiked water samples.

Spiked mancozeb (μM)	Determined concentration (μM)
1.0	2.00 ± 0.55
10.0	6.08 ± 1.57
50.0	9.62 ± 1.55

As mancozeb is widely used in agricultural industries, Mn(II) released from mancozeb accumulates in terrestrial environments. Thus, measuring the potential of the biosensors to quantify Mn(II) in soil is essential. Accordingly, residual Mn(II) was determined from the mancozeb-amended soil samples, which were prepared by spiking 5.0, 10.0, and 100.0 mg of mancozeb into 1 g of loam soil. Subsequently, Mn(II) was extracted with 10 mL of distilled water. The residual Mn(II) in the soil leachates was analyzed using *E. coli* cell-based biosensors and ICP–AES. Table 4 summarizes the corresponding results. We assumed that majority of mancozeb was degraded in the soils as the soils were incubated with water for 3 days. Unlike the biosensors directly spiked with mancozeb, the amount of Mn(II) increased proportionally with the amount of mancozeb. In the case of 10 mg of mancozeb-spiked soil sample, 12.2 ppm of Mn(II) determined using the *E. coli* biosensor was converted to 122 μg of Mn(II) because the samples were extracted with 10 mL of distilled water. Notably, this amount did not reflect the total Mn(II) amount released from mancozeb because some amount of Mn(II) was absorbed by the soil. Moreover, the Mn(II) amounts detected through the biosensor assay and ICP–AES were comparable, with the measurement accuracy of the biosensor assay being >90%. This proved the potential and applicability of the newly generated biosensors in monitoring Mn(II) in environmental systems.

**TABLE 4:** The amount of Mn(II) originated from mancozeb amended soil samples determined by *Escherichia coli*-cell based biosensor and inductively coupled plasma–atomic emission spectroscopy (ICP–AES) analysis.

Spiked mancozeb (mg/g of soil)	Soil leachate		Accuracy (%)
	*E. coli* biosensor (ppm)	ICP-AES (ppm)	
5.0	12.20 ± 2.55	13.3	91.7
10.0	22.34 ± 1.42	24.5	91.2
100.0	205.21 ± 7.64	226.7	90.5

## 4 Discussion

Heavy metal(loid)s, which are used in several industries, are released into environmental systems and threaten human health (Duruibe et al., 2007; Tchounwou et al., 2012). Thus, the levels of heavy metal(loid)s in environmental systems should be regularly monitored. Several technique-based sensors, including electrochemical, nanomaterial, biochemical, and transcription factor-based sensors, have been developed to analyze heavy metals (He et al., 2021; Naresh and Lee, 2021). Particularly, bacterial cell-based biosensors have been widely used because of their simplicity and convenience compared with other analytical instruments (Sørensen et al., 2006; Mahr and Frunzke, 2016).

Although bacterial cell-based biosensors can target diverse chemicals, antibiotics, and heavy metals, their basic working mechanisms are similar. Typically, the genetic systems that respond to stress have been employed as sensing domains that initiate the transcription of reporter domains (Fernandez-López et al., 2015; Pham et al., 2022). For example, the promoter and TF pairs such as P*_*tetO*_/tetR* for tetracycline, P*_*padC*_/padR* for *p*-coumaric acid, P*_*zntA*_/zntR* for cadmium, P*arsA/.arsR* for arsenic were used for the biosensor systems. When the reporter genes were fused with each promoter region, the expression of reporter proteins were induced by the interaction between TFs and target materials.

In this study, we developed Mn(II)-sensing bacterial cell-based biosensors using a genetic system related to Mn(II) homeostasis and a strategy to enhance selectivity and sensitivity by molecular engineering. The *mnt* regulon, including *mntS*, *mntP*, *mntH*, and *mntR* as components in *E. coli*, can maintain the cellular levels of Mn(II) (Patzer and Hantke, 2001; Waters et al., 2011; Sun et al., 2012; Martin et al., 2015). Accordingly, *mntH* and *mntS* encoding an Mn importer and an uncharacterized small protein, respectively, were induced by low Mn(II) levels, and *mntP* encoding an Mn exporter was activated by high Mn(II) levels. Therefore, plasmids carrying promoter regions of *mntS/mntH* and *mntP* fused with *egfp* were constructed and transformed into *E. coli* to verify the Mn(II)-sensing capability of the biosensors.

As shown in Figure 1, *E. coli* cell-based biosensors were developed by introducing sensing plasmids, including pMntH-eGFP, pMntS-eGFP, pMntP-eGFP, and pMntP-ribo-eGFP, and exposing them to Mn(II). However, only P*_*mntS*_:egfp* responded to the Mn(II) exposure (Figure 2D). Biosensors harboring P*_*mntH*_:egfp* did not exhibit any fluorescent signal upon Mn(II) exposure (Figure 2A). Although P*_*mntP*_:egfp* and P*_*mntP–ribo*_:egfp* showed fluorescent signals, the signals did not respond to Mn(II) concentrations (Figures 2B, C), implying that MntP transcription was not regulated by MntR alone. Moreover, the riboswitch in the promoter region of MntP could have played a role in transcription, along with MntR.

As the Mn(II)-sensing biosensors were developed based on the *mnt*-operon regulated by MntR, the roles of MntR were identified. The protein-regulating function of MntR may have controlled the transcription of *mntS*. Furthermore, the plasmid carrying *mntSp:egfp* was introduced into *E. coli* BL21-WT and *mntR* cells and exposed to Mn(II). The signal changes of biosensors lacking *mntR* did not change upon Mn(II) exposure, whereas the biosensors based on *E. coli* WT showed decreased eGFP expression (Figure 4). Moreover, Mn(II) responses were restored when the recombinant *mntR* was introduced, indicating that the MntR is a regulatory protein that represses the transcription of *mntS* and responds reversibly to Mn(II) concentration. Conclusively, this was consistent with the mechanisms of the *mnt* regulon reported previously, and the assumption of generating reverse-responding Mn(II) sensors using the promoter of *mntS* was accurate (Patzer and Hantke, 2001; Waters et al., 2011).

Our previous studies indicated that the sensitivity of biosensors was enhanced by deleting genes related to target exportation, such as *copA* and *zntA* (Kang et al., 2018a,b). Such deletions of heavy metal exporters cause cellular accumulation of target compounds, resulting in the target sensitivity of biosensors. Similarly, *mntP*-deficient *E. coli* was generated and used as a host strain. As shown in Figure 5, the responses of *E. coli* cell-based biosensors toward Mn(II) were enhanced by two times after deleting *mntP*. Thus, MntP deletion caused Mn(II) accumulation inside the cells, indicating that MntP plays a major role in Mn(II) exportation in *E. coli*, and that the properties of bacterial-cell-based biosensors can be modulated by genetic engineering of host strains.

Although *E. coli* cell-based biosensors employing P*_*mntS*_*:*egfp*, and genetic engineering of the host strain showed sensitivity toward Mn(II), target selectivity requires further verification. As described above, the selectivity of the transcription factor-based biosensors was determined by the selectivity of the regulatory protein, MntR. If the metal(loid) ions bound to MntR and induced the conformational changes, the transcription level of reporter genes under P*_*mntS*_* would be changed. Therefore, biosensors based on pMntS-eGFP were exposed to various heavy metal(loid) ions and their subsequent responses were analyzed to verify the Mn(II) selectivity. As shown in Figure 6, fluorescence signal changes were observed with about five of induction coefficient value from Mn (II) exposure, while others showed less than 1.5 of induction coefficient values. This result indicated the biosensor was selective to only Mn(II) among the tested metal(loid)s; thus, proving that the *E. coli* cell-based biosensors employing P*_*mntS*_* was applicable to quantify Mn(II).

As the newly generated biosensors showed Mn(II) specificity and sensitivity, the application of biosensors to environmental systems was examined. Accordingly, water and soil samples amended with Mn(II) and mancozeb were prepared, and the residual Mn(II) amount was quantified. The biosensors were directly subjected to the prepared water samples, and the fluorescence intensities were subsequently measured; moreover, the soil samples were applied as soil extracts. Mn(II) concentration in the Mn(II)-spiked water samples was determined using a standard curve, and the resultant accuracy of the biosensor assay was >90% (Table 2). The Mn(II) concentration in the mancozeb-containing water samples was quantified through a biosensor assay (Table 3). However, the amount of Mn(II) was not proportional to the amount of mancozeb, which could be attributed to the degradation rate of mancozeb in water (López-Fernández et al., 2016, 2017). Unlike the water samples, the mancozeb-amended soil samples were incubated with water for 3 days during sample preparation. According to previous reports, an incubation period of 3 days is sufficient for mancozeb degradation to ethylenethiourea (ETU), although the degradation rates varied depending on the experimental conditions, such as pH, temperature, and treatments (Lehotay and Kisova, 1993; Hwang et al., 2002; López-Fernández et al., 2017). Further, the amount of Mn(II) in the soil leachate was determined by a biosensor and ICP–AES, and the amount of Mn(II) was found to increase proportionally with the amount of spiked mancozeb (Table 4). Importantly, the amount of Mn(II) determined by the biosensor assay was similar to that determined by ICP–AES, with over 90% recovery. The biosensor developed based on *mntS* was a new Mn(II)-specific biosensor that could effectively quantify Mn(II) in environmental systems. Although the biosensor showed the capability to quantify Mn(II) in environmental samples, it should be considered the applicability to the harsh conditions of samples. If the growth of biosensors were not inhibited by samples, the environmental samples could be applied to biosensors. Since *E. coli* was used as host cells, the samples containing materials to inhibit the growth of biosensors could not be analyzed. However, it could be solved by further sample preparation processes.

In conclusion, new Mn(II)-specific biosensors were developed and their applicability to environmental systems was verified. Because Mn is relatively less toxic than other toxic heavy metals, Mn has not been monitored extensively in environmental systems. This poses a serious problem because the industrial application of Mn has rapidly increased. Further, Mn-conjugated fungicides have been widely used in agricultural industries, and their toxic effects have been reported. Thus, the Mn(II)-specific *E. coli* cell-based biosensor developed in this study serves as a valuable tool for monitoring and assessing the risks of Mn in different environmental systems.

## Data availability statement

The datasets presented in this study can be found in online repositories. The names of the repository/repositories and accession number(s) can be found in the article/Supplementary material.

## Author contributions

YJ: conceptualization, investigation, and data analysis. YL: investigation and data analysis. YK and CP: investigation. HC and GJ: conceptualization and writing. YY: supervision, data analysis, and writing. All authors contributed to the article and approved the submitted version.
