# Supplementary material for: Development of novel Escherichia coli cell-based biosensors to monitor Mn(II) in environmental systems
Source: Front Microbiol. 2022 Dec 19;13:1051926. doi: 10.3389/fmicb.2022.1051926 (PMC9806134; doi:10.3389/fmicb.2022.1051926)

*Supplementary Data for:*

Development of novel *Escherichia coli* cell-based biosensors to monitor Mn(II) in environmental systems

**Yangwon Jeon^1^, Yejin Lee^1^, Yeunhong Kim^1^, Chanhee Park^1^, Hoon Choi^2^, Geupil Jang^3*^, Youngdae Yoon**^1*^

^1^Department of Environmental Health Science, Konkuk University, Seoul 05029, Republic of Korea

^2^Department of Life and Environmental Sciences, Wonkwang University, Iksan 54538, Republic of Korea.

^3^School of Biological Sciences and Technology, Chonnam National University, Gwangju 61186, Republic of Korea

*** Correspondence:**Geupil Jang
yk3@chonnam.ac.kr

Youngdae Yoon
yyoon21@gmail.com

Keywords: *E. coli*-based biosensor, Mn(II) monitoring, MntR transcription factor, *mnt*-operon

**Table S1. The lists of primers used for plasmid construction**

| **Name** | **Sequence (5’-3’)** |  |
| --- | --- | --- |
| mntP promoter  mntP-ribo  mntS promoter  mntH promoter  MntR  MntR K73L  MntR R77L  MntR H135A | AT*AGATCT*GGCTGTTAATCCGCGTTC  AA*TCTAGA*AAAACACATCATCGCCCC  AT*AGATCT*GGCTGTTAATCCGCGTTC  AA*TCTAGA*AAATGAGGGCGCAACCTT  AC*AGATCT*TCGTGCGCACATTGTAAAC  CC*TCTAGA*TCCTTGAGGGATGATTGCAT  CC*AGATCT*TCAGAGAAATCACCACAATC  CC*TCTAGA*GCCTCTAAAACATAGCCTTT  AT*CATATG*AGTCGTCGCGCAGGTAC  TA*CTCGAG*TCATTTGGCACCGTGTT  CAACCGACGGTGGCTCTAATGCTTAAGCGGC  GCCGCTTAAGCATTAGAGCCACCGTCGGTTG  CTAAAATGCTTAAGCTGCTGGCAACCATGG  CCATGGTTGCCAGCAGCTTAAGCATTTTAG  GCGGAAGGCATGGAGGCGCATGTTAGTGAAGAG  CGCCTTCCGTACCTCCGCGTACAATCACTTCTC | forward  reverse  forward  reverse  forward  reverse  forward  reverse  forward  reverse  site-directed mutagenesis  site-directed mutagenesis  site-directed mutagenesis |

^*^ The underlined letters on primer sequences were indicated the restriction enzyme sites.

**Figure S1. The effects of MntR in *E. coli* cell-based biosensors.** The biosensor strains on LB agar plates were illuminated under UV light. (A) *E. coli* WT with pMntS-eGFP, (B) *E. coli-mntR* with pMntS-eGFP, (C) *E. coli-mntR* with pMntS-eGFP and pCDF-MntR WT


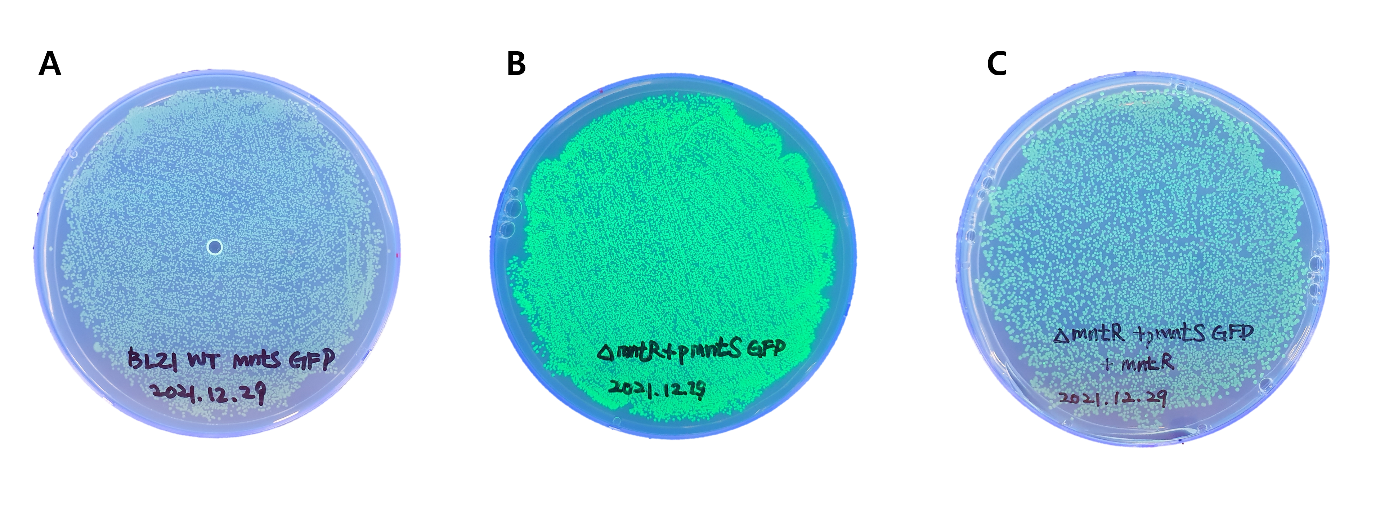

Supplement: Supplementary file 1 [file Data_Sheet_1.docx]
